# Aberrant Immune Features after Recovery from COVID-19 in Patients with Systemic Lupus Erythematosus and Other Autoimmune Diseases

**DOI:** 10.3390/biomedicines11102807

**Published:** 2023-10-17

**Authors:** Siyue Yu, Hao Li, Kai Zhang, Gong Cheng, Yifan Wang, Yuan Jia, Linchong Su, Yuebo Jin, Miao Shao, Jing He

**Affiliations:** 1 Department of Rheumatology and Immunology, Peking University People’s Hospital, Beijing 100044, China; pkuysy2000@163.com (S.Y.); lihao_0306@163.com (H.L.); zk569975536@126.com (K.Z.); chenggong@bjmu.edu.cn (G.C.); w7103012341@163.com (Y.W.); jiayuan1023@bjmu.edu.cn (Y.J.); xianglifuer@126.com (Y.J.); shaomzw@163.com (M.S.); 2 Hubei Provincial Key Laboratory of Occurrence and Intervention of Rheumatic Diseases, Hubei Minzu University, Enshi 430074, China; linchongsu@126.com

**Keywords:** COVID-19, pandemic influenza, immunology mechanisms, broad immune response, regulatory T cell

## Abstract

Considering the large number of individuals who have already been infected and may have reinfection, the post-infection effects of COVID-19 are of great importance for clinical practice and predicting disease trends. However, our understanding of the potential long-term effects, particularly on immunity, after recovering from COVID-19 remains limited. The aim of this study was to investigate the abnormal immunological factors that contribute to the prolonged immunological effects of COVID-19. Two groups of patients were enrolled in the study, including 11 individuals with various autoimmune diseases (AIDs) and 16 patients diagnosed with systemic lupus erythematosus (SLE). Detailed clinical symptoms were closely monitored, and peripheral mononuclear cells were analyzed using flow cytometry. The clinical status was evaluated using the SLE Disease Activity Index (SLEDAI) and the Clinical Global Impressions (CGI) index. The proportions of follicular T helper cells (Tfh) exhibited significant increases in both cohorts (AID: *p* = 0.03; SLE: *p* = 0.0008). Conversely, the percentages of Foxp3^+^ and CD4^+^ regulatory T cells (Treg) were reduced in patients following COVID-19 infection (AID: *p* = 0.009, 0.05, resp.; SLE: *p* = 0.02, 0.0009, resp.). The percentages of Th2 and Th17 cells were significantly increased in SLE patients (*p* < 0.05). Exacerbated conditions were observed in SLE patients two months after infection (SLEDAI, *p* < 0.05). Our findings show that COVID-19 infection increases Tfh cells and decreases Treg cells in patients of AIDs, worsening pathogenetic immune status in post-recovery populations.

## 1. Introduction

In the years following the start of the coronavirus disease 2019 (COVID-19) pandemic, our understanding of the clinical manifestations, lingering symptoms, and laboratory features of this disease has significantly improved. Although the majority of infected individuals experience mild symptoms, COVID-19 can manifest as a systemic disease that impacts multiple organ systems [1]. It is now widely recognized that some patients who have had previous SARS-CoV-2 infection may experience long-term consequences during or after their recovery from COVID-19 [2], a condition now referred to as post-acute sequelae of COVID-19 [3,4,5]. Patients with autoimmune diseases seem to experience more severe COVID-19 illness [6,7] and have an increased risk of hospitalization and readmissions across various disease categories following COVID-19 infection [8,9,10]. However, our understanding of the potential long-term effects, particularly on immunity, after recovery remains limited.

COVID-19 shares similarities with autoimmune diseases in terms of clinical manifestations, immune response, and disease pathogenesis. SARS-CoV-2 utilizes the host’s machinery to replicate and to evade the immune response [11], and viral proteins become similar to host proteins because of this process. It is thought that the virus is able to mimic host proteins, leading to the easy production of autoantibodies as the immune system fights the infection. The development of both autoimmune diseases and COVID-19 is influenced by processes such as molecular mimicry, hyperstimulation, and dysregulation of the immune system [12].

As a result, treatments used for autoimmune diseases have been widely employed in severe cases of COVID-19 [13]. Specific autoantibodies have been detected in COVID-19 patients [14,15]. Furthermore, there are reports of individuals developing autoimmune diseases following COVID-19 infection, with manifestations ranging from organ-specific conditions (e.g., immune thrombocytopenic purpura [16] and Guillain–Barré syndrome [17]) to systemic autoimmune and inflammatory conditions (e.g., systemic vasculitis [18], systemic lupus erythematosus [19], and rheumatoid arthritis [20,21]). Some researchers estimate the risk of autoimmune diseases rises about 2 months after COVID-19 infection [22,23,24], which may be attenuated by COVID-19 vaccination [24]. These observations have sparked interest in further exploring the relationship between COVID-19 and autoimmune diseases [25,26].

The aim of this study is to elucidate the prolonged immunological effects of COVID-19 in two cohorts: one comprising patients with various autoimmune diseases (AIDs) and another consisting of individuals with systemic lupus erythematosus (SLE) only. By observing changes in the immune spectrum and clinical symptoms, we can discover why patients are at risk for developing or exacerbating autoimmune diseases after infection. This investigation may help optimize management strategies for patients with SLE and other autoimmune diseases. Additionally, it may provide insights into post-acute sequelae of COVID-19 and autoimmune diseases potentially induced by COVID-19 infection.

## 2. Materials and Methods

### 2.1. Study Patients

We recruited a total of 27 patients with both COVID-19 and autoimmune diseases at Peking University People’s Hospital from November 2022 to May 2023 and divided them into two groups. The AID group consisted of 11 patients with various autoimmune diseases, and the SLE group included 16 patients with SLE. The recruitment period for both groups of patients was relatively concentrated.

The AID group consisted of 11 patients, including 4 patients with SLE (one of whom also had antiphospholipid syndrome, APS), 3 patients with Sjogren’s syndrome (SS), 1 patient with rheumatoid arthritis (RA), 1 patient with Bechet’s disease (BD), 1 patient with mixed connective tissue disease (MCTD), and 1 patient with dermatomyositis (DM). All of these patients had been diagnosed with an autoimmune disease for a minimum of 6 months prior to COVID-19 infection. All patients survived COVID-19, though the severity and manifestations of the disease varied. Three patients in the AID group received vaccines in February, March, and August 2021, while the others were not vaccinated. All of them had been diagnosed with an autoimmune disease before vaccination, and the symptoms of COVID-19 in these patients were relatively mild.

### 2.2. COVID-19 Infection and Data Collection

COVID-19 status and confirmation of infection were determined by a series of COVID-19 PCR tests performed by the Clinical Laboratory at Peking University People’s Hospital. Positive test results and dates were extracted from both cohorts. In both groups, most of the patients who tested positive had mild symptoms. Demographic and clinical characteristics were collected at the time of cohort entry. In the AID group, data were collected before COVID-19 infection and approximately two weeks after infection when patients had almost recovered. In the SLE group, data were collected before COVID-19 infection and two months after infection.

### 2.3. Clinical, Laboratory, and Immunological Assessments

Lymphocytes and T, B, and NK cell subgroups, as well as the proportions of different T cell subtypes (including regulatory T cells and follicular helper T cell subsets), were evaluated. Other related laboratory parameters, such as immunoglobulin levels and complement levels, were also assessed by the Clinical Laboratory at Peking University People’s Hospital. Clinical global impression (CGI) and systemic lupus erythematosus disease activity index (SLEDAI) scores were evaluated independently by two independent physicians [27,28]. Immunological analysis of peripheral blood mononuclear cells was performed before COVID-19 infection and after recovery from infection. The counts and proportions of lymphocytes and T, B, and NK cell subgroups were analyzed using flow cytometry with a FACS Calibur flow cytometer (BD Biosciences, Franklin Lake, NJ, USA). The proportions of regulatory T (Treg) cells, follicular helper T (Tfh) cells, and other T subgroups were analyzed using flow cytometry with an FACS Aria II flow cytometer (BD Biosciences, Franklin Lake, NJ, USA). The FlowJo software package (v.10.6.2; Tree Star, BD Biosciences) was used to analyze the data. Lymphocytes were defined with FSC/SSC and CD45^+^; T cells were defined as CD3^+^; CD4^+^ T cells were defined as CD3^+^CD4^+^; CD8^+^ T cells were defined as CD3^+^CD8^+^; B cells were defined as CD3^−^CD19^+^; and NK cells were defined as CD3^−^CD16^+^CD56^+^. Tfh cells were defined as CD3^+^CD4^+^CXCR5^+^PD1^high^; CD4^+^ Treg cells were defined as CD3^+^CD4^+^CD25^high^CD127^low^; Foxp3^+^ Treg cells were defined as CD3^+^CD4^+^CD25^high^Foxp3^+^; CD161^+^ Treg cells were defined as CD3^+^CD4^+^CD25^high^ CD127^low^CD161^+^; CLA^+^ Treg cells were defined as CD3^+^CD4^+^CD25^high^CD127^low^ CLA^+^; Naïve Th cells were defined as CD4^+^CD45RA^+^; and T effector (Teff) cells were defined as CD4^+^CD25^−/+^Foxp3^−^. Th1 cells were defined as CXCR3^+^CCR6^−^CD45RA^−^CD3^+^CD4^+^; Th2 cells were defined as CXCR3^−^CCR6^−^CD45RA^−^CD3^+^CD4^+^; and Th17 cells were defined as CXCR3^−^CCR6^+^CD45RA^−^CD3^+^CD4^+^.

### 2.4. Statistical Analysis

All statistical analyses were performed using R (v.4.2.2; R Foundation for Statistical Computing, Vienna, Austria). Continuous data were analyzed using t-tests or Mann–Whitney U tests based on normal distribution. Continuous variables were presented as median with quartiles, and categorical variables were presented as percentages. A *p*-value less than 0.05 was considered statistically significant. Outliers were removed using the 3σ method.

## 3. Results

### 3.1. Patient Demographics and Clinical Characters

Baseline demographic and clinical characteristics of the patients are summarized in Table 1. Of the 11 AID patients, the majority received concomitant steroids (prednisone 5–50 mg/day), and 5 were taking hydroxychloroquine (HCQ; 400 mg/day). Regarding current immunosuppressive agents, three patients were on mycophenolate mofetil (MMF, 1000–1500 mg/day), three were on tacrolimus (TAC, 1–3 mg/day), two were on cyclosporin A (CsA, 100–150 mg/day), and one was on cyclophosphamide (CTX, 400 mg every two weeks).

After receiving treatment, the patients’ COVID-19-related symptoms improved, and all patients recovered from a serious infection based on medical records. Clinical laboratory tests showed some significant changes when comparing values before and after COVID-19 recovery, as shown in Table 2. Similarly, in the SLE group, baseline characteristics are presented in Table 1, and changes in laboratory parameters are summarized in Table 2.

### 3.2. Changes in Total T, B, and NK Cells after COVID-19 Infection

Changes in immune cell profiles before and after recovery from COVID-19 were examined in both the AID and SLE groups. In the AID group, a slight increase in NK cell counts (*p* = 0.04) and proportions (*p* = 0.13) was observed after COVID-19 recovery, while the proportion of CD4^+^ T cells decreased significantly (*p* = 0.05). In the SLE group, total B cell counts (*p* = 0.03) and proportions (*p* = 0.05) were reduced after recovery. No significant differences were found in total lymphocyte counts between baseline and post-recovery data for either group (Table 3).

### 3.3. The Imbalance between Tregs and Teff Cells still Present in Patients with COVID-19 Recovery

To further investigate the underlying mechanism, we analyzed Tfh/Treg subpopulations using flow cytometry. In the AID group, the proportion of Tfh cells was significantly elevated (*p* = 0.033), particularly in patients with SLE. Treg cells were diminished in almost all eleven patients (Foxp3^+^ Treg, *p* = 0.009; CD4^+^ Treg, *p* = 0.053), suggesting that SARS-CoV-2 infection disrupts immune tolerance and potentially leads to broken tolerance. The decreased Treg/Teff ratio highlights this condition (Foxp3^+^ Treg, *p* = 0.019). Proportions of CD161^+^ Treg cells showed a slight decrease (*p* = 0.033). Similar patterns were observed in the SLE group, with increased Tfh cells (*p* = 0.0008) and decreased Treg cells (Foxp3^+^ Treg, *p* = 0.02; CD4^+^ Treg, *p* = 0.0009). The Treg/Teff and Treg/Th17 ratios were also reduced (Foxp3^+^ Treg, *p* = 0.009, 0.002, respectively). The proportions of CD161^+^ Treg cells exhibited a slight decrease (*p* = 0.03). These findings indicate a continued imbalance between Tregs and Teff cells in patients with COVID-19, even two months after recovery (Table 4).

### 3.4. Proportion of Th1, Th2, Th17 Cells Differed among Autoimmune Diseases

Analysis of Th1/Th2/Th17 cells revealed varying changes depending on the specific autoimmune disease. In the SLE group, pronounced changes were observed, with significant increases in Th2 (IL4, *p* = 0.00001) and Th17 (IL17, *p* = 0.0003) cells. Higher Th1 (*p* = 0.10) was also observed. This suggests that COVID-19 could worsen certain autoimmune conditions, like SLE, over time (Table 4).

### 3.5. Autoimmune Disease Activity Increased in Patients following COVID-19 Recovery

**Figure 1:**
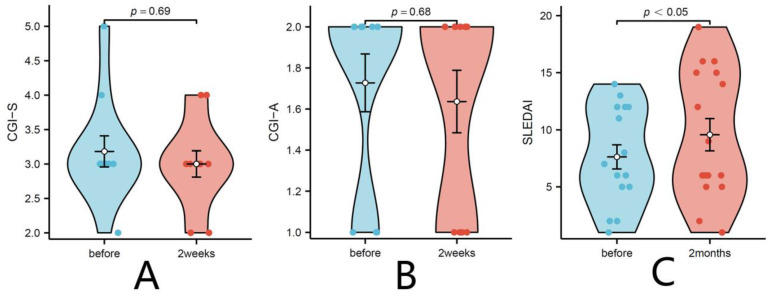
Changes in CGI-S, CGI-A, and SLEDAI before COVID-19 and post-recovery. (**A**,**B**) Changes in CGI-S (**A**) and CGI-A (**B**) in AID patients before and after COVID-19. (**C**) Changes in SLEDAI in SLE patients before and after COVID-19. The statistical analysis was determined by Wilcoxon matched-pairs signed-rank test. Differences with *p* < 0.05 were considered significant. CGI-S, clinical global impressions severity; CGI-A, clinical global impressions activity; SLEDAI, systemic lupus erythematosus disease activity index; before: before COVID-19 infection; 2weeks: 2 weeks after COVID-19 infection; 2months: 2months after COVID-19 infection.

To assess disease activity, the CGI severity (CGI-S) and SLEDAI scores were used in the AID and SLE group, respectively. There was no significant change in CGI-S (*p* = 0.69) and CGI-A (*p* = 0.68) scores two weeks after infection, indicating stable disease states due to ongoing clinical care. However, approximately 2 months later, new symptoms emerged in half of the patients (Table 5), leading to increased SLEDAI scores (*p* = 0.04). These results are depicted in Figure 1.

## 4. Discussion

Considering the large number of individuals who have already been infected, the post-infection effects of COVID-19 are of great importance for clinical practice and predicting disease trends. However, viral contact and infection are highly unpredictable in autoimmune patients, which complicates the reporting of long-term effects of COVID-19 in this population. In this study, we aimed to examine the differences between the pre-COVID-19 infection and post-recovery states in patients with SLE and other autoimmune diseases.

Previous studies reported that lymphocyte count decreases significantly during COVID-19 infection [29]. After recovery, antibody levels were found to negatively correlate with time since viral contact, while cellular immunity showed a persistent relationship even 20 months later [30]. As part of the defensive strategy, long-term immunological memory is stored in the form of memory T cells. Regardless of disease severity, specific memory T cell responses are maintained in COVID-19 convalescent patients for up to 10 months post-symptom onset [31]. These memory T cells remain ready to elevate the immune response against the pathogen by eliciting new effector cells. Additionally, Tfh cells are crucial for enhanced B cell response as pathogens are constantly evolving and attempting to elude antibody recognition [32]. At the same time, because viral proteins are similar to host proteins, the molecular mimicry mechanism of autoantigens serves to drive the chronic autoimmune process.

In our study, there were minimal changes in total lymphocyte counts before infection and after recovery. Medical records suggest that the patients in our study have largely recovered from severe COVID-19 infection. One notable difference observed in the AID group was a substantial decrease in the proportion of CD4^+^ T cells, suggesting persistent immune hyperreactivity. Specifically, circulating Tfh cells exhibited a marked increase, indicating elevated proinflammatory factors. Moreover, the suppressive Treg cells were significantly reduced, potentially leading to increased inflammation. These findings suggest that COVID-19 further disrupts immune tolerance in patients with autoimmune diseases [33], which has important implications for their clinical practice. The significant decrease in the Treg/Teff and Treg/Th17 ratio after recovery parallels the characteristics observed in SLE patients [34]. This suggests that COVID-19 infection may induce or exacerbate SLE and other autoimmune diseases by complicating the underlying pathogenic conditions [35].

We also recruited a separate SLE group and extended the observation period to approximately 2 months. The changes in T cell subgroups observed in this SLE group were consistent with but more pronounced than those in the AID group, indicating that the effects of COVID-19 continued to exacerbate over time. These results provide further evidence that the alterations were not merely transient physiological changes during acute infection. Furthermore, some patients, particularly those with severe conditions, developed new symptoms. In summary, COVID-19 appears to exacerbate the underlying disease conditions in some AID patients, especially those with SLE.

Healthcare providers should carefully consider the optimal approach to therapy adjustment to achieve better outcomes in patients with autoimmune diseases and a history of COVID-19. Although patients with autoimmune diseases are at higher risk of developing COVID-19 and severe COVID-19 [36], chronic glucocorticoid usage is not associated with worse COVID-19 outcomes [37]. Providers should advise patients to avoid abrupt withdrawal of drugs even after acquiring SARS-CoV-2 infection and to continue treatment with the appropriate doses [38]. We recommend a 1-month post-discharge follow-up visit to monitor disease status and adjust medications under professional guidance.

Furthermore, the benefits of COVID-19 vaccination outweigh the potential risks in this population. Published guidelines recommend vaccination for autoimmune patients in stable disease periods to reduce the risk of severe infection, and according to experts, the benefits of COVID-19 vaccination for AID patients outweigh the potential risk for new-onset autoimmunity [39]. In this study, only three patients in the AID group received vaccines in February, March, and August 2021. The symptoms of COVID-19 in these patients are relatively mild, but the number of cases is small, and the length of time between vaccination and infection makes it difficult to evaluate the effect of the vaccine on the patient’s infection in this study.

The findings presented in this study offer valuable insights into the immune imbalances that can occur in individuals recovering from COVID-19. The observed changes in the immune spectrum help explain why patients are at increased risk for developing or exacerbating autoimmune diseases around 2 months post-infection [22,23,24]. These results highlight the need for continued monitoring and appropriate medical intervention for autoimmune patients during the subacute phase after COVID-19. Additionally, further exploration of immunomodulatory treatments is warranted to address the pathogenic effects of aberrant immune responses following SARS-CoV-2 infection. Understanding the longitudinal impacts of COVID-19 on immune function will be key to improving outcomes and managing autoimmune risks. There is a need for future investigations into therapeutic immunological approaches to mitigate complications in the post-viral period.

The main limitations of this study are the relatively small number of patients and the variability in background therapies, which make it difficult to definitively attribute the observed effects solely to the Tfh response alone. Therefore, conducting a larger study with more extensive patient data would deepen our understanding of the disease course and clarify the implications of these results. Further research focused on the effects of COVID-19 on patients with specific autoimmune diseases would provide valuable insights into the role of COVID-19 in these populations. Standardizing treatment regimens within study groups could help isolate the impact of COVID-19 infection on Tfh and other immunological responses. Larger patient samples would also allow analysis of differences between mild, moderate, and severe COVID-19 infection on long-term autoimmune disease progression. Overcoming these limitations will require multi-center collaborative studies into the lasting effects of COVID-19 on autoimmunity.

## 5. Conclusions

In conclusion, this study found that COVID-19 infection increases Tfh cells and decreases Treg cells in patients of SLE and other AIDs. These immunological changes suggest that COVID-19 infection disrupts immune tolerance and aggravates pathogenetic immune responses. Furthermore, the data indicate that COVID-19 infection may induce new onset of AIDs, such as SLE, in post-recovery populations. These findings may help rheumatologists recognize high-risk patients and take appropriate management.

## Figures and Tables

**Table 1:** Baseline characteristics of patients with autoimmune diseases combined with COVID-19.

Demographic and Clinical Characteristics	AID Group (*n* = 11)	SLE Group (*n* = 16)
Sex (female), *n* (%)	8 (72.73)	14 (87.50)
Age, median (IQR *)	54 (52.25, 59.50)	55 (41.75, 59.50)
Disease duration (year), median (IQR)	10 (5.25, 10.25)	15 (10.25, 18.50)
**Clinical symptoms, *n* (%)**		
Rash	4 (36.36)	7 (43.75)
Oral ulceration	3 (27.27)	2 (12.50)
Dry mouth	4 (36.36)	3 (18.75)
Arthritis	4 (36.36)	4 (25)
Alopecia	2 (18.18)	4 (25)
Fever	1 (9.09)	4 (25)
Muscle pain	2 (18.18)	0
Fatigue	3 (27.27)	4 (25)
Headache	0	3 (18.75)
Platelet reduction	3 (27.27)	3 (18.75)
**Treatment, *n* (%)**		
Glucocorticoid	11 (100)	16 (100)
HCQ	5 (45.45)	9 (61.25)
MMF	3 (27.27)	11 (73.75)
CTX	1 (9.09)	1 (6.25)
CsA	2 (18.18)	0
LEF	0	1 (6.25)
TAC	3 (27.27)	2 (12.50)

* IQR, interquartile range. Abbreviations: HCQ, hydroxychloroquine; MMF, mycophenolate mofetil; CTX, cyclophosphamide; CsA, cyclosporin a; LEF, leflunomide; TAC, tacrolimus.

**Table 2:** Changes in laboratory parameters before COVID-19 and post-recovery.

	AID Group (*n* = 11)			SLE Group (*n* = 16)		
Routine Laboratory Tests, Median (IQR *)	Before COVID-19	Post-Recovery	*p*-Value	Before COVID-19	Post-Recovery	*p*-Value
WBC (×10^9^/L)	4.60 (3.25,6.35)	4.20 (3.40,4.55)	0.482	5.77 (4.03,6.60)	5.50 (3.95,6.50)	0.883
HGB (g/L)	105.00 (93.50,134.00)	117.00 (93.00,130.50)	0.766	124.00 (113.00,132.50)	124.50 (116.50,131.75)	0.700
PLT (×10^9^/L)	131.00 (57.50,199.50)	129.00 (61.50,151.00)	0.116	181.00 (149.75,191.75)	178.00 (160.00,206.00)	0.437
Lymphocytes (×10^9^/L)	0.80 (0.61,0.95)	0.70 (0.49,0.95)	0.188	0.97 (0.83,1.38)	1.05 (0.63,1.43)	0.094
Neutrophil (×10^9^/L)	3.10 (1.98,4.95)	3.00 (2.50,3.44)	0.920	3.40 (2.64,4.38)	3.60 (2.65,4.48)	0.493
ALT (g/L)	21.00 (16.50,30.50)	14.00 (11.50,20.50)	0.052	14.00 (12.00,21.00)	13.00 (12.00,21.00)	0.918
AST (g/L)	23.00 (21.50,29.00)	21.00 (14.00,23.00)	0.084	16.00 (14.00,18.00)	19.00 (14.00,21.00)	0.463
Serum creatinine (μmol/L)	58.00 (55.00,84.00)	57.00 (53.50,88.00)	0.799	64.00 (54.00,76.00)	62.00 (56.00,78.00)	0.948
Albumin (g/L)	36.00 (33.45,41.80)	39.60 (36.45,40.70)	0.220	39.00 (35.20,42.00)	40.10 (35.50,40.70)	0.531
Uric acid (μmol/L)	321.00 (230.00,404.50)	292.00 (228.50,344.50)	0.611	281.00 (240.00,346.00)	285.00 (260.00,316.00)	0.686
Urea (mmol/L)	6.45 (4.02,7.18)	6.00 (3.45,7.05)	0.333	5.12 (3.41,6.60)	5.70 (5.00,6.70)	0.322
CRP (mg/L)	0.70 (0.50,2.90)	1.90 (0.50,17.70)	0.129	0.50 (0.50,1.20)	0.50 (0.50,2.40)	0.096
ESR (mm/h)	15.00 (6.50,27.50)	26.00 (10.50,48.00)	0.321	5.00 (2.00,7.00)	8.00 (4.00,15.00)	0.037
C3 (g/L)	0.79 (0.59,0.93)	0.97 (0.50,1.10)	0.115	0.84 (0.76,0.92)	1.02 (0.98,1.12)	1.43 × 10^−5^
C4 (g/L)	0.15 (0.10,0.22)	0.22 (0.13,0.44)	0.079	0.22 (0.19,0.26)	0.30 (0.29,0.40)	0.0009
IgA (g/L)	2.23 (1.41,5.10)	2.54 (1.23,4.00)	0.355	1.85 (1.48,2.09)	1.56 (1.34,1.78)	0.057
IgG (g/L)	13.85 (9.23,19.27)	9.12 (8.42,12.48)	0.186	8.30 (6.90,10.80)	7.56 (5.87,9.15)	0.146
IgM (g/L)	1.26 (0.80,1.95)	1.09 (0.63,1.47)	0.145	0.43 (0.19,0.79)	0.43 (0.25,0.54)	0.085

* IQR, interquartile range. Abbreviations: ALT, alanine transaminase; AST, aspartate aminotransferase; C3, complement 3; C4, complement 4.

**Table 3:** Changes in lymphocyte and its subgroups before COVID-19 and post-recovery.

	AID Group (*n* = 11)			SLE Group (*n* = 16)		
Flow Cytometry Tests, Median (IQR *)	Before COVID-19	Post-Recovery	*p*-Value	Before COVID-19	Post-Recovery	*p*-Value
lymphocyte (/μL)	922.00 (744.00,1333.50)	878.00 (630.00,1094.00)	0.424	1155.00 (960.00,1604.50)	1172.00 (759.50,1481.00)	0.504
T(/μL)	791.00 (649.50,887.00)	656.00 (521.50,766.50)	0.323	960.00 (722.00,1500.50)	904.00 (593.50,1299.00)	0.507
T(%)	77.10 (70.45,85.20)	75.00 (64.95,82.25)	0.694	85.00 (76.85,88.15)	79.22 (76.03,89.98)	0.644
CD4^+^ T(/μL)	410.00 (301.00,606.50)	329.00 (186.50,500.00)	0.227	425.00 (379.50,653.50)	370.00 (238.50,662.00)	0.583
CD4^+^ T (%)	48.30 (36.70,52.40)	38.20 (30.00,56.05)	0.045	38.90 (32.25,50.20)	37.80 (29.70,47.07)	0.328
CD8^+^ T (/μL)	239.00 (173.50,442.00)	264.00 (179.50,303.50)	0.870	476.50 (274.00,761.25)	347.50 (293.75,536.75)	0.113
CD8^+^ T (%)	27.10 (17.40,40.80)	27.90 (18.05,45.10)	0.556	43.30 (28.25,47.05)	43.53 (29.74,48.00)	0.800
CD4/CD8	1.78 (1.11,2.66)	1.22 (0.87,3.05)	0.282	1.12 (0.70,1.51)	0.90 (0.64,1.54)	0.601
B(/μL)	76.50 (27.25,107.25)	40.50 (28.25,58.50)	0.107	56.00 (22.00,85.00)	26.00 (4.00,71.00)	0.031
B(%)	7.90 (3.10,14.00)	4.30 (3.35,9.05)	0.256	4.50 (2.35,9.10)	3.10 (0.15,5.05)	0.052
NK(/μL)	84.00 (39.50,106.00)	100.00 (48.50,238.50)	0.039	92.00 (34.50,192.00)	98.00 (52.00,166.50)	0.549
NK (%)	7.30 (4.85,10.85)	10.00 (6.25,18.15)	0.125	7.80 (4.70,11.55)	10.60 (5.98,16.15)	0.708

* IQR, interquartile range.

**Table 4:** Changes in pTfh/Treg and Th1/2/17 subgroups before COVID-19 and post-recovery.

	AID Group (*n* = 11)			SLE Group (*n* = 16)		
Flow Cytometry Tests, Median (IQR *)	Before COVID-19	Post-Recovery	*p*-Value	Before COVID-19	Post-Recovery	*p*-Value
Tfh (%)	1.60 (1.15,2.45)	3.40 (2.10,5.15)	0.033	2.10 (1.50,2.68)	3.80 (2.40,5.33)	0.0008
CD4^+^ Treg (%)	8.90 (3.18,15.73)	6.70 (4.10,8.75)	0.053	11.40 (7.58,16.53)	7.05 (5.25,8.23)	0.0009
Foxp3^+^ Treg (%)	8.02 (5.96,8.87)	4.63 (3.50,7.14)	0.009	8.64 (7.51,11.56)	7.08 (5.74,8.25)	0.017
CD161^+^ Treg (%)	14.20 (9.80,19.30)	7.10 (5.60,11.20)	0.033	11.40 (5.98,16.48)	6.65 (3.60,9.45)	0.033
CLA^+^ Treg (%)	12.60 (9.53,19.23)	8.80 (3.18,15.73)	0.386	14.85 (8.95,17.08)	13.70 (7.25,27.10)	0.334
Naïve Th (%)	47.50 (24.13,53.02)	34.74 (16.72,46.74)	0.230	31.19 (12.78,38.78)	22.38 (15.35,37.97)	0.710
Teff (%)	90.98 (90.43,92.47)	93.96 (91.23,94.83)	0.105	87.88 (86.24,90.23)	89.72 (89.14,92.14)	0.060
Foxp3^+^ Treg/Teff	0.082 (0.062,0.096)	0.049 (0.036,0.076)	0.019	0.097 (0.086,0.129)	0.078 (0.057,0.083)	0.009
Th1 (%)	11.90 (10.18,17.73)	10.55 (7.98,17.35)	0.467	18.40 (9.35,22.30)	20.43 (16.25,22.88)	0.104
Th2 (%)	2.16 (1.78,2.31)	2.11 (1.36,2.46)	0.800	1.45 (1.00,1.81)	2.08 (1.91,2.50)	1.10 × 10^−5^
Th17 (%)	1.62 (0.80,2.22)	1.70 (1.16,1.88)	0.785	1.24 (1.01,1.52)	1.71 (1.68,2.15)	0.0003
Foxp3^+^ Treg/Th17	5.37 (3.77,9.57)	2.97 (2.39,5.90)	0.478	6.63 (5.45,11.29)	3.46 (2.91,4.74)	0.002

* IQR, interquartile range.

**Table 5:** New symptoms in SLE patients 2 months after infection.

Clinical Symptoms	Psychosis	Arthritis	Myositis	Headache	Fever	Proteinuria	Leukopenia
Patient 1	○						
Patient 2							
Patient 3							
Patient 4		○					
Patient 5					○		
Patient 6							
Patient 7				○	○		
Patient 8							
Patient 9						○	
Patient 10							○
Patient 11							
Patient 12			○				
Patient 13						○	
Patient 14							
Patient 15							
Patient 16							

Note: The “○” means that the patient in the corresponding row has the symptom in the corresponding column. These symptoms were dictated by the patients and confirmed by corresponding examinations and the physicians.

## Data Availability

The data presented in this study are available on request from the corresponding author.
